# Microbiologically Influenced Corrosion of Copper and Its Alloys in Anaerobic Aqueous Environments: A Review

**DOI:** 10.3389/fmicb.2022.806688

**Published:** 2022-04-04

**Authors:** Roberta Amendola, Amit Acharjee

**Affiliations:** Mechanical and Industrial Engineering Department, Montana State University, Bozeman, MT, United States

**Keywords:** copper, biofilm, marine environment, potable water systems, microbial corrosion (MIC)

## Abstract

Regardless of the long record of research works based on microbiologically influenced corrosion (MIC), its principle and mechanism, which lead to accelerated corrosion, is yet to be fully understood. MIC is observed on different metallic substrates and can be caused by a wide variety of microorganisms with sulfate-reducing bacteria (SRB) being considered the most prominent and economically destructive one. Copper and its alloys, despite being used as an antimicrobial agent, are recorded to be susceptible to microbial corrosion. This review offers a research overview on MIC of copper and its alloys in anaerobic aqueous environments. Proposed MIC mechanisms, recent work and developments as well as MIC inhibition techniques are presented focusing on potable water systems and marine environment. In the future research perspectives section, the importance and possible contribution of knowledge about intrinsic properties of substrate material are discussed with the intent to bridge the knowledge gap between microbiology and materials science related to MIC.

## Introduction

Corrosion is a naturally occurring physiochemical process where pure metal and/or alloys undergo a chemical change from ground state to an ionized state due to transfer of electrons from metal to an external electron acceptor. Corrosion progresses in a series of redox reactions (anodic oxidation and cathodic reduction) accompanied by hydrolysis which is often responsible for changing the pH of the local environment and causing the deterioration of the material. This deterioration can be guided by microorganisms when they attach to the metal surface and form a cohesive unit called biofilm consisting of microbial cells and extracellular polymeric substances (EPS). This phenomenon is known as microbiologically influenced corrosion (MIC) or biocorrosion (Beech and Gaylarde, 1999; Chen et al., 2014; Jia et al., 2019; Little et al., 2020).

The annual cost of damage due to corrosion is estimated to be around 2.5 trillion USD globally and MIC is considered to be responsible for approximately 20% of the total cost and also for 50% of all pipeline infrastructure failures (Javaherdashti, 1999; Wang et al., 2014; Liu et al., 2016). MIC causes immense financial loss in many industries such as oil and gas production, transportation, aircraft, marine, nuclear, chemical, power generation, civil engineering, medical implants, etc. (Brauer et al., 2017; Talha et al., 2019; Dou et al., 2020).

Due to its detrimental impact on the economy, MIC has been the topic of extensive research for the past few decades. In spite of the long record of works, the fundamental interactions between various microorganisms and metals in an electrolytic medium are still not fully understood (Blackwood, 2018). Combating MIC has proven to be difficult as it can take place as an independent corrosion mechanism and is also capable of co-existing with other mechanisms. MIC has been linked to a broad spectrum of microorganisms, both aerobic and anaerobic. Bacteria, algae, protozoa, diatom, archaeon, and fungi are members of that spectra of organisms (Blackwood, 2018). Bacteria are regarded as the primary contributor to MIC failures and as a result a major portion of the research work on MIC is based on the influence of bacterial biofilms on different engineering metals and their alloys. Sulfate-reducing bacteria (SRB; Hamilton, 1985), sulfur-oxidizing bacteria (SOB; Estokova et al., 2011), manganese-oxidizing (MOB) bacteria (Dickinson and Lewandowski, 1996), iron-oxidizing bacteria (IOB), iron-reducing bacteria (IRB; Obuekwe et al., 1981), and bacteria secreting organic acids and exopolymers (Beech et al., 1991) are the ones that typically co-exist in a naturally originated biofilm structure (Beech and Sunner, 2004). Among these microorganisms SRB, which are found in a broad range of anaerobic environmental conditions (e.g., shallow marine and freshwater sediments, soil and deep subsurface environments), were found to be responsible for almost half of all MIC related cases (Lee et al., 1995; Yuan et al., 2013).

Copper and copper alloys are widely used in many applications such as domestic and industrial pipeline system, heat exchanger, fire sprinkler system, structures for marine environment, etc., because of their superior machinability, thermal and electrical conductivity, ease of soldering, and corrosion-resistant properties due to patina formation on the outer surface of copper which causes passivation enhancing corrosion resistance in oxygen (Wagner and Little, 1993; Chen et al., 2014; Güngör et al., 2015).

Despite having a long record of being used as an antibacterial and chemical control agent for microorganisms, copper and its alloys are prone to microbial corrosion (Trevors and Cotter, 1990). Many MIC cases have been reported for copper and its alloys and are often related to catastrophic failures of the infrastructure (Javed et al., 2016). Due to its wide application as infrastructure material, better understanding of copper MIC is highly valuable.

This review presents an overview on recent works and developments in the field of MIC of copper and its alloy as well as on inhibition techniques focusing on potable water systems and marine environment.

## Mechanisms of Copper Microbiologically Influenced Corrosion

Microbiologically influenced corrosion can occur under both aerobic and anaerobic conditions. When the environment lacks free oxygen, it can be defined as “anaerobic.” Water reduction is then required to supply cathodic current necessary to support and continue the corrosion reaction. When the dissolved oxygen concentration is enriched enough to carry on the corrosion reaction without the necessity of water reduction, the environment can be defined as “aerobic” (Blackwood et al., 2004; Blackwood, 2018). It must be noted that these definitions are not universal but rather metal-specific as the same environment can occur as aerobic to the active metals but anaerobic to the passive metals.

Microbiologically influenced corrosion in anaerobic conditions is regarded as the more financially detrimental variety. To date, extensive research has been conducted to elucidate the underlying MIC mechanism particularly for ferrous substrate. Gu et al. (2009) classified anaerobic MIC attacks into two different types (Gu, 2012). Type I, also known as extracellular electron transfer MIC (EET-MIC) caused by the respiration of microbes, and Type II, defined as metabolite MIC (M-MIC) caused by the secretion of corrosive metabolites. During EET-MIC, microbes harvest energy by extracting electrons from elemental iron and transporting them into the cytoplasm to reduce an electron acceptor like sulfate. Unlike EET-MIC, M-MIC does not depend on a biofilm’s need for energy, as metabolites like protons, organic acids and sulfides oxidize metals extracellularly causing the development of a surface film.

In the recent review by Blackwood (2018), the author states that the direct electron transfer theory is incorrect. Considering the process from the electrochemistry point of view, single-step charge transfer at the electrode/electrolyte would not occur over a distance larger than 2 nm when aqueous species are involved (Gray and Winkler, 2005). During EET-MIC, the minimum distance to cover is 7.5 to 10 nm corresponding to the cell’s membrane thickness (Hine, 2005). Such length increases to hundreds of nanometers when the electron participates in the cell metabolism by reaching the mitochondria or the cell nucleus. This distance is too large for a single electron transfer step, so the direct electron transfer mechanism is improbable and need further evidence to be supported.

During M-MIC, the crystalline structure, chemical composition, and adherence to the substrate of the surface film depend on the conditions under which it forms. When the film is well adhered and insulating, it may also protect the metal from further corrosion (Shoesmith et al., 1980; Zheng et al., 2015; Dou et al., 2018; Jia et al., 2018; Wang et al., 2020).

Copper is not easily corroded as it is relatively noble, so SRB are unable to directly extract its electrons for sulfate reduction; therefore, it is unlikely that the EET-MIC mechanism would apply.

Recent studies support M-MIC as the most probable corrosion mechanism for copper (Javed et al., 2016; Dou et al., 2018; Wang et al., 2020). Puigdomenech and Taxen (2000) identified reaction (1) between copper and H_2_S as thermodynamically favorable:


(1) $$2\,C\,u\,\left(s\right)+H_{2}\,S\,\left(g\right)\to {\mathrm{Cu}}_{2}\,S\,\left(s\right)+H_{2}\,\left(g\right)$$

Bard et al. (1985) proposed the following anodic (1a) and cathodic (1b) reactions:


(1a) $$2\,C\,u\,\left(s\right)\to 2\,C\,u^{2+}+2\,e^{-}$$

(1b) $${\mathrm{HS}}^{-}+H^{+}+2\,e^{-}\to S^{2-}+H_{2}$$

The amount of H_2_S produced by SRB is locally high under the biofilms, causing a decrease in the local pH. This theory is supported by Webster et al. (2000) and Davidson et al. (2009) who propose that such pH decrease at the interface copper/solution is the main factor controlling MIC. However, the production of H_2_S by SRB is indirect, as the final metabolic product is HS^–^, which then combines with H^+^ to form H_2_S. This way the local environment would be H^+^ depleted leading to an increase in pH, not a decrease as expected (Elliott et al., 1998; Blackwood, 2018; Dou et al., 2018). As results are contradictory, additional investigation is needed to elucidate the underlying mechanism of copper M-MIC. The authors of this review suggest that future research should also account for environmental conditions like temperature and pressure as these parameters can change the proposed reactions’ equilibria as well as the nature of the developed surface layer.

Models proposed to explain copper MIC suggested that EPS is responsible for creating preferential cathodic sites because of its cation-selective characteristics (Siedlarek et al., 1994; Wagner et al., 1997). EPSs produced by different bacteria appeared to have different abilities to bind copper ions and induce different copper oxidation states. Increased deposition of EPS was related to bacteria overcoming the copper toxicity by binding the ions in the polymer. This binding phenomenon leads to the formation of copper ions concentration cells and the generation of a weakly acidic environment (Geesey et al., 1986; Angell and Chamberlain, 1991; Chen and Zhang, 2018).

It was proposed that biofilm produced acidic metabolites may be associated with high copper by-product release (Davidson et al., 2009) while EPSs are thought to be responsible for copper pitting by heterogeneously accumulating on the surface, causing localized dissolution of copper (Geesey et al., 1986; Jolley et al., 1988; Bremer and Geesey, 1991; Geesey, 1991; Wagner et al., 1997; Chen and Zhang, 2018).

Several studies in the last few years have shown that nutrients can greatly impact the corrosion behavior of SRB (Javed et al., 2015; Dou et al., 2018, 2019; Liu et al., 2018; Salgar-Chaparro et al., 2020). Wang et al. (2020) found that riboflavin could enhance the MIC of carbon steel induced by SRB but had no effect on copper MIC. Chen et al. (2022) compared the effect phosphorous lack, nitrogen lack, and lack of both nutrients with total nutrition conditions. It was found that decreased nutrition in culture medium led to lower planktonic and sessile cell count, reduced H_2_S concentration, and looser EPS. These recent results highlighted the fact that the availability of nutrients in the environment or in experimental conditions impact bacterial metabolic pathways; therefore, some of the mechanisms proposed earlier may vary or no longer apply.

## Microbiologically Influenced Corrosion of Copper in Potable Water System

Owing to its advantageous characteristics like corrosion resistance and antiseptic properties, copper has been widely used in drinking water distribution systems for decades (Keevil, 2004; Lee et al., 2012; Luo et al., 2017; Vargas et al., 2017). However, enhanced localized corrosion, which subsequently resulted in failure of the infrastructure, has been observed. This issue was linked to the growth of biofilms on copper pipes, which was also found responsible for increased metal concentration in drinking water (Bremer et al., 2001; Beech, 2004; Reyes et al., 2008; Kip and van Veen, 2014). These phenomena have been reported as an expensive problem in countries around the world like Scotland, Germany, England, Saudi Arabia, and United States (Keevil, 2004).

Enhanced biofilm growth was observed under certain operational conditions, particularly when the environment pH is in the range 6.1–6.3 and for long-term or periodic stagnation of water in the pipe. Microbial activities originate several copper mobilization and immobilization processes like precipitation, uptake, sorption, and intracellular accumulation, however. The intensity of these processes depends on the type of microbial community present in the biofilm and the water physicochemical conditions (Vargas et al., 2017). Better knowledge of the process responsible for enhanced corrosion in potable water pipes and of the copper transportation mechanisms are of utmost importance to ensure healthy water quality.

Traditional laboratory experiments conducted to investigate MIC mostly consider stagnant conditions to evaluate copper release; however, this approach presents a major weakness as it does not account for how the hydrodynamic flow, naturally present in potable water systems, affects the release of copper. Werner investigated systems subjected to different flow-stagnation cycles and suggested that copper dissolution occurred under both flow and stagnation conditions, but only during stagnation copper ions are transported by diffusional phenomena and accumulated until an equilibrium with the solid by-products formed on the pipe walls is reached (Werner, 1995).

Pizarro and Vargas (2016) studied copper release phenomena in actual household drinking water affected by MIC and laboratory system consisting of copper pipes supplied by synthetic water. The ageing time (e.g., biofilm development) of the tested pipes was different being, respectively, 2 years for the household system and 4 weeks for the laboratory one. Data were collected under the ideal plug flow assumption. Tested pipes were flushed at a constant laminar flow rate following the methodology presented by Calle et al. (2007) and Vargas et al. (2010). Under the plug flow assumption, the mass of copper released during flushing experiments was three times (laboratory) to nine times (household) the mass in the bulk water before the tap was opened. The authors attributed this difference to pipes’ ageing time which defines the development of the complex surface system comprised of biofilm, EPS, and corrosion byproducts. This well agreed with a previous study by the authors (Pavissich et al., 2010) where it was found that the community developed in the mature biofilm present in the field seems to be more adapted to high copper concentrations.

To address the difference in the observed copper release, the authors developed a long-term (350 days) mathematical model which considered the following processes: (a) growth and decay of the biofilm on the inner surface of the pipe; (b) transport of chemical species, by diffusion, during stagnation time; (c) transport of chemical species, by convection, during laminar flow conditions; (d) speciation of copper; and (e) release of copper ions into the liquid. The model succeeded in reproducing the experimental copper release behavior confirming that complexation of copper on the biomass is of importance. It also emphasized the importance of considering hydrodynamic flow conditions and highlighted that diffusion transport is not the main mass transport mechanism. On the other hand, one of the assumptions of the model was that the release of copper from the complex surface system during flow occurs instantly. There is not enough information regarding the kinetics of this phenomenon; therefore, further investigation is needed.

Recently, Galarce et al. (2020) also performed experiments following the methodology proposed by Vargas et al. (2010) on an actual premise plumbing system. The results obtained by the authors suggested that the composition of biofilms and the corrosion rate were variable throughout the sampling time. No clear relationship among the biofilm age, the corrosion rate, and the released amount of copper was observed. This investigation confirmed the need for studying copper MIC of potable water pipelines as a dynamic phenomenon that cannot be characterized by a single aging time or short-term extrapolation.

Based on the presented discussion of published work, the authors of this review believe that the current major challenge for potable water systems is the inability to develop an accurate MIC predictive model. Potable water distribution networks are designed for liquid velocities of about 0.2–0.5 m/s, and hydrodynamic conditions can range from laminar to turbulent along with stagnant waters (Manuel et al., 2007); hence, an accurate model should account for the proper effect of hydrodynamics at the metal-biofilm-water interfaces. Previous studies showed that the morphology, distribution, resistance, and adherence characteristics of the biofilm are flow-dependent (e.g., patchy structures or elongated streamers) (Stoodley et al., 1998; Percival et al., 1999; Hall-Stoodley et al., 2004).

This highlights the need for more comprehensive models that, in addition to the already accounted for conditions, would consider some, if not all the following variables:

- electrochemical reactions generated by the interaction between pre-existing or developed surface layers with the surrounding environment.
- kinetics of different copper ion species.
- transient or turbulent flow.
- detachment of corrosion or biofilm particles due to shear stresses.
- environmental temperature and/or pressure.
- biofilm morphology and distribution.

## Microbiologically Influenced Corrosion of Copper in Marine Environment

Copper and copper alloys are extensively used in the marine environment for the fabrication of ships, components of marine structures like ship piping system, heat exchangers, and also for offshore infrastructure like power plants (Carvalho et al., 2014). In seawater, these materials quickly develop a well-adherent biofilm over the surface which generally presents a heterogeneous composition (Geesey et al., 1986; Zuo, 2007; Zhang et al., 2019a). Under the biofilms the presence of reduced pH, organic acids, sulfide, and acidic polysaccharides can compromise the protective passive layer causing severe corrosion (Wagner and Chamberlain, 1997). Geochemical factors of the marine environment such as pH (Ponsonnet et al., 2008), nutrients (Sheng et al., 2008), salinity (Briand et al., 2017), and temperature (Witt et al., 2012) are essential in defining microbial surface colonization.

Carvalho et al. (2014) performed microbiological and molecular analysis of the biofilm formed on copper alloys exposed to sea water. Results revealed that the bacterial composition was similar to others found in water bodies of different parts of the world. Recently, Zhang et al. (2019a) used a similar approach to characterize in situ marine microbial communities developed on various alloys along with the microorganisms growing at different depths in corrosion layers, to determine the potential corrosion-causing microorganisms. Both works concluded that specific metal or metallic alloy influences the bacteria selection that causes the development of a unique microbial biocenosis.

Zhang et al. performed metagenomic analysis on copper-alloy surfaces exposed to natural marine environment and found that the developed biofilm possesses a unique microbial and functional gene composition. This study provided the first evidence of the genetic potential of copper-resistance and MIC mechanisms in mature biofilms. It was discovered that the two predominant functional genes identified in copper-associated biofilms, stress response *rpoE* gene, and possible copper transporter *ABC.CD.P* gene are significant to microbial communities in natural formed biofilms to control heavy metal homeostasis. These two genes coexist with *cus* and *cop*-encoding copper-efflux systems, implying that mature biofilms have the great genetic potential to tolerate copper ions (Zhang et al., 2019b). The present study further showed that field studies on marine biofilms using omics-approaches are needed to complement laboratory investigations on MIC caused by a single species or its biofilms.

Fuel biodegradation linked to sulfate reduction can lead to corrosion of the metallic infrastructure in marine environments. Military ships often use seawater compensated fuel ballast tanks. As the ship engines use fuel, seawater is pumped aboard to compensate for weight and volume losses. Seawater-compensated fuel ballast systems are designed as a series of tanks, typically interconnected by sluice pipes made of a copper-nickel alloy (Liang et al., 2018) wherein fuel directly contacts seawater (Lyles et al., 2013; Liang et al., 2016), increasing the possibility of anaerobic conditions and biocorrosion.

The biological stability of emerging biofuels and their potential impact on copper–nickel alloys has not been well documented. Using a recently published protocol (Liang and Suflita, 2015), Liang et al. (2018) conducted an experiment to test and compare alternative and petroleum derived fuels’ biological stability and their impact on MIC of copper–nickel alloy. As the introduction of marine sediment to the ballast tanks in the field is unavoidable (Burkholder et al., 2007) the authors incorporated marine sediments into the seawater collected from San Diego bay which are known to contain anaerobic hydrocarbon degrading sulfate reducing microorganisms (Coates et al., 1997). The authors used 70/30 copper-nickel alloy as substrate material, two alternative biofuels (Camelina-JP5 and FT-F76), and their respective petroleum-derived counterparts (F76 and JP5). JP5 and Camelina-JP5 lead to the highest corrosion rates, respectively, 0.89 ± 0.05 mpy and 0.94 ± 0.22 mpy. Much lower corrosion rates were associated with the F76 and FT-F76 namely 0.22 ± 0.08 mpy to 0.55 ± 0.17 mpy. These findings collectively reported the tendency of the fuel toward accelerating biogenic sulfide production consequently impacting MIC of copper-nickel alloy in seawater.

The studies presented in this section emphasized the heterogeneous composition of the natural marine biofilm. To recognize the contribution of different microorganisms and characterize the interactions among those groups in microbial corrosion processes is a rather complex and difficult task. Monocultures are still used in most of the laboratory studies on microbial corrosion inhibition.

The authors of this review suggest that future laboratory studies on marine MIC should examine the synergistic/antagonistic effect of multi-species biofilms on copper corrosion behavior (Batmanghelich et al., 2017; Phan et al., 2021). The use of genomics analyses on natural marine biofilm should also be considered as a powerful complementary addition to laboratory experiments.

## Recent Developments in Inhibition of Copper Microbiologically Influenced Corrosion

### Triazoloazepinium Bromide Biocide

Triazoloazepinium bromide (TAB) has been known to inhibit growth activity of SRB, IRB, denitrifying bacteria, and ammonifying bacteria (Kurmakova et al., 2018). In their recently published work, Kurmakova et al. (2018) showed that TAB inhibited the sulfate-reducing activity of bacteria by increasing the redox potential of the corrosive medium by up to 170 mV. The authors proposed that TAB adsorbs on copper by transferring electrons from negative ions present in its molecule, to the unoccupied metal orbitals. The biofilm is then formed on the adsorbed TAB layer with a loose structure not corrosively aggressive. The rate of copper MIC in the tested water-salt medium with bacterial sulfate reduction decreased by a factor of 11.7, which corresponds to an MIC inhibition efficiency of 93.5%.

### 1,2,3-Benzotriazole, 5-(3-aminophenyl) Tetrazole as Inhibitors and Tetrakis (hydroxymethyl) Phosphonium Sulfate as Biocide

As biocides have low efficiency against biofilms along with the potential of being harmful to the aquatic environments, alternate approaches to inhibit MIC are needed (Lambert and Johnston, 2001; Bridier et al., 2011). One such strategy is using a combination of an inhibitor and a biocide at a very low concentration to combat both MIC and general corrosion. MIC of 90/10 copper–nickel alloy caused by SRB in marine condition has been shown to be successfully decreased by two new formulations containing, BTAH and APT as inhibitors and THPS as biocide. Chaitanya Kumar and Appa Rao (2016) presented that both of the following formulations: (1) 3.33-mM BTAH + 0.245-mM THPS and (2) 6.5-mM APT+ 0.245-mM THPS showed inhibition efficiency greater than 99%. Potentiodynamic polarization results work showed that both formulations shift the corrosion potential to more positive values and decreased the corrosion current density. In particular, when formulation (1) was used, microbial count studies underlined the absence of SRB cells and no corrosion products were observed on the surface, even after 90 days of immersion in seawater. The inhibitor and biocide concentrations used in this study follow the guidelines established by the Organization for Economic Co-Operation and Development (OECD; Painter, 1995). Hence, both BTAH and THPS are not expected to exhibit adverse effects on aquatic environments. Further research is needed to verify the safety of the proposed formulation and to establish successful protocols for larger-scale applications.

### Commercial Corrosion Inhibitor and Bacteria Control Agent

Commercial corrosion inhibitor KURITA, S-7653 composed of polyphosphate, phosphate, zinc salts, and a polymer and bacteria control agent KURITA, F-5100 were used in the study by Li et al. (2018) to mitigate MIC of pure copper in cooling water systems in the field and in simulated laboratory experiments. It was reported that the corrosion rate was reduced from 0.0177 mm/year in the control system to 0.0021 mm/year with the use of the inhibitor. Weight loss data for the combined action of the corrosion inhibitor and the bacteria control agent resulted in inhibition efficiency of about 88%.

### 2D Graphene and Hexagonal Boron Nitride Coatings

Graphene is a carbon allotrope consisting of a single layer of bonded carbon atoms arranged in a hexagonal lattice. hBN has a honeycomb structure like graphene, consisting of covalently bonded boron (B) and nitrogen (N) atoms. Typical thickness of 2D atomic monolayer structures is ∼0.4 nm for both materials (Santos et al., 2021). Graphene and hBN are characterized by a reduced lattice size (∼0.3 nm) which, being smaller than the bacteria and their metabolites, acts as an ion impermeable barrier (Parra et al., 2015, 2017). Graphene and h-BN coatings on copper extensively suppressed interaction between substrate and bacteria (Parra et al., 2015; Chilkoor et al., 2018). The coatings proved to be advantageous in both directions, as bacterial community was protected from copper toxicity and substrate was protected from MIC. hBN coatings are expected to be effective at suppressing galvanic effects due to their insulating nature, unlike graphene, where the local defects, such as layer discontinuities, can accelerate the corrosion process because of the strong cathodic nature of graphene with respect to copper, causing galvanic coupling (Chilkoor et al., 2018; Wu et al., 2019). However, the fact that single-layer insulating h-BN and conducting graphene were equally effective to prevent contact of bacteria with the copper emphasizes that charge transfer through these 2D coatings is unlikely (Parra et al., 2015). Chilkoor et al. (2018) and Al-Saadi et al. (2021) suggested that ability of 2D coating to inhibit SRB colonies development on nickel-copper alloy is instead to be mainly attributed to their hydrophobic properties.

According to the safe piped water recommendations by the World Health Organization (WHO), chlorine, chloramines, and chlorine dioxide are the three disinfectants that have been, and are still, widely used to successfully control microbial growth in potable water lines (Ainsworth, 2004). As a careful assessment of the short and long-term effects on human health of any alternative chemical compound would need to be conducted, the authors do not foresee a near future change in this approach.

The use of 2D coatings is a promising and environment-friendly solution for MIC inhibition; nevertheless, such approach is still in its infancy when compared to biocides use.

2D-coating antibacterial properties strongly depend on the coating morphology which is defined by the manufacturing method. Currently, chemical vapor deposition (CVD) is the most suitable technique to produce atomically smooth 2D coatings with low defects and high reproducibility. However, CVD is a rather expensive technique as it requires high temperature, accurate control of operative parameters, and post-production transfer processes, *via* wet etching, which limit the coated surface size. Also, most deposition processes have been performed on prepared flat surfaces, which do not well reflect the configuration of materials deployed in the field (e.g., circular pipes). Further research is needed to optimize CVD manufacturing processes and to identify suitable alternative approaches for developing appropriate and durable 2D coatings to be applied on the larger scale and complex geometries.

## Future Research Perspectives

Microbiologically influenced corrosion has been widely accepted as a complex phenomenon influenced by microbial and environmental variables. Innovative approaches are needed to better replicate operational conditions which are able to reproduce the corrosion morphological features at the rate observed in the field.

While surveying the current trends in copper MIC research, the authors of this review noticed that two scarcely addressed areas can be identified:

- the metrics for consistent characterization and comparison of corrosion processes.
- the effect of metal intrinsic properties on corrosion, namely, grain structure, surface finish and presence of a native oxide layer.

From the authors’ perspective, the lack of attention to the above listed areas presents a considerable weakness in MIC investigations. The importance of such topics and why they should be addressed as part of the MIC investigation protocol is discussed below.

### Corrosion Characterization Metrics

Dou et al. (2018) and Wang et al. (2020) highlighted the importance of quantifying corrosion in a more comprehensive fashion. Copper samples presented rather severe damage where substrate thickness was lost due to uniform corrosion. In such MIC case, if only pitting were to be considered, the corrosion severity would have been largely underestimated because pit depth would have been measured from the corroded surface rather than from the uncorroded one. Therefore, both pit depth and weight loss must be used to describe corrosion severity. Dou et al. (2018) used relative pitting severity (RPS) to reflect the relative importance of pitting corrosion to uniform corrosion. RPS is defined as the ratio of pit growth rate to uniform corrosion rate based on specific weight loss, and can be calculated from the following equation (2):


(2) $$R\,P\,S=\frac{a\,v\,e\,r\,a\,g\,e\,m\,a\,x\,i\,m\,u\,m\,p\,i\,t\,d\,e\,p\,t\,h\,x\,m\,e\,t\,a\,l\,d\,e\,n\,s\,i\,t\,y}{a\,v\,e\,r\,a\,g\,e\,s\,p\,e\,c\,i\,f\,i\,c\,w\,e\,i\,g\,h\,t\,l\,o\,s\,s}$$

RPS should be used along with pit depth data or weight loss data to describe corrosion severity adequately as follows:

- RPS ≫ 1, pitting corrosion is much more severe than uniform corrosion.
- RPS ≈ 1, pitting corrosion and uniform corrosion are equally important.
- RPS ≪ 1, uniform corrosion is much more severe than pitting corrosion.

### Effect of Metal’s Intrinsic Properties

#### Grain Structure

Most metallic materials are manufactured through a series of thermomechanical processes (e.g., rolling followed by annealing). The grain structure is the result of such processes and is responsible for determining the material mechanical and chemical properties. During mechanical processing, the material is plastically deformed. The stresses applied during deformation cause an increase in the dislocation (linear defect in the crystal structure) density within the grain structure. Locations with higher dislocation density are characterized by residual stresses which raise the grain structure internal energy and, in turn, increase the material chemical reactivity. Higher dislocation density results in the preferential attack of grain of relatively high internal energy which leads to higher localized corrosion susceptibility (Robin et al., 2012; Zhou et al., 2013; Ma et al., 2015; Zhang et al., 2017).

The stresses introduced through plastic deformation processes and the consequent higher dislocation density can be reduced by thermal treatments like annealing. It is therefore expected that a properly heat-treated material will present lower internal energy and it will therefore be less prone to corrosion.

Zhao et al. (2014) and Yuan et al. (2016), showed that incorporation of engineered grain structure containing low internal energy features achieved through different thermo-mechanical treatments enhanced the overall corrosion resistance of copper. This should be considered as a promising approach to combat copper MIC.

Characterization of the material grain structure with microscope techniques and/or advanced tools like electron backscatter diffraction (EBSD) which can also map residual stresses distribution, should be included in the MIC investigation protocol as it can provide important preliminary knowledge about how the material will react with the surrounding environment.

#### Surface Finishing

A material surface finishing is, in most cases, defined by the surface roughness which represents the measure of the finely spaced micro-irregularities on the surface texture (e.g., surface grooves produced by grinding). Surface roughness plays an important role on the corrosion behavior of metals. Li and Li (2006) reported that an increase in the surface roughness of copper increases the pitting susceptibility and general corrosion rate. On average, for metals able to develop a passive layer like copper, a decrease in surface roughness increases the corrosion resistance while for the ones with no passive layer (e.g., mild steel), a reverse trend has been observed (Burstein and Pistorius, 1995; Abosrra et al., 2009; Alvarez et al., 2010). Rough surfaces offer a larger interfacial area to the corrosive environment; hence, an enhanced corrosion rate is expected. Also, surface irregularities may create micro-reaction sites promoting pit growth by hosting a very corrosive environment, preferential local dissolution of the substrate, and by trapping corrosion products (Burstein and Pistorius, 1995; Zuo et al., 2002). A surface with decreased roughness (smoother) reduces the occurrence of localized corrosion phenomena by reducing the number of micro-reaction sites capable of being activated.

In addition to influencing the corrosion behavior of the metallic substrate, surface roughness was found to be a key factor in determining the extent of bacterial colonization which, in turn, determines the biofilm development and attachment characteristics (Crawford et al., 2012).

Microbiologically influenced corrosion laboratory experiments are commonly performed on a metallic substrate with a surface finishing of 400, 600, or 1200 grit. The authors believe that, for the correct evaluation of corrosion processes, and for consistent comparison, the initial surface roughness to be considered for laboratory experiments should be selected to be as similar as possible to the one employed in the material’s operative condition. An evaluation of the material’s surface morphological features through 2D or 3D profilometry before the start of laboratory experiments is highly encouraged.

#### Presence of a Native Oxide Layer

Several metallic materials, including copper and copper-nickel alloys, when exposed to air, naturally develop a thin, hard, and relatively inert surface layer, usually an oxide (known as the “native oxide layer” or “passive layer”) that protects the material from further corrosion. The native oxide layer can form during or after the manufacturing process and it will most likely persist when the material is deployed in the operative conditions. The interaction between biofilm and native layer of the metallic substrate (electrochemical behavior) is expected to be different when compared to the bare substrate. As a consequence, changes in the mechanisms presented in section “Mechanisms of Copper Microbiologically Influenced Corrosion” of this review may be observed.

For this reason, accurate characterization of the native oxide layer in terms of composition, morphology, and thickness should be performed prior to laboratory experiments. Substrate polishing is usually performed to offer a “fresh” surface during MIC laboratory experiments. The authors recommend that, along with polished samples, “as-received” (unpolished) samples should be tested for better representing the material in actual working conditions.

Undisputedly, for MIC to be properly investigated, a multi and interdisciplinary approach is required. This should include collaborative efforts across several scientific areas, namely, microbiology, materials science, engineering, solid-state physics, and electrochemistry.

## Author Contributions

Both authors listed have made a substantial, direct, and intellectual contribution to the work, and approved it for publication.

## Conflict of Interest

The authors declare that the research was conducted in the absence of any commercial or financial relationships that could be construed as a potential conflict of interest.

## Publisher’s Note

All claims expressed in this article are solely those of the authors and do not necessarily represent those of their affiliated organizations, or those of the publisher, the editors and the reviewers. Any product that may be evaluated in this article, or claim that may be made by its manufacturer, is not guaranteed or endorsed by the publisher.
